# A dose-response relationship of smoking with tuberculosis infection: A cross-sectional study among 21008 rural residents in China

**DOI:** 10.1371/journal.pone.0175183

**Published:** 2017-04-06

**Authors:** Haoran Zhang, Henan Xin, Xiangwei Li, Hengjing Li, Mufei Li, Wei Lu, Liqiong Bai, Xinhua Wang, Jianmin Liu, Qi Jin, Lei Gao

**Affiliations:** 1 MOH Key Laboratory of Systems Biology of Pathogens, Institute of Pathogen Biology, and Centre for Tuberculosis, Chinese Academy of Medical Sciences and Peking Union Medical College, Beijing, China; 2 Jiangsu Provincial Center for Diseases Control and Prevention, Nanjing, China; 3 Hunan Provincial Institute of Tuberculosis Prevention and Control, Changsha, China; 4 Gansu Provincial Center for Diseases Control and Prevention, Lanzhou, China; 5 The Sixth People’s Hospital of Zhengzhou, Zhengzhou, China; Fudan University, CHINA

## Abstract

**Objectives:**

China has high burden on both of tuberculosis (TB) and tobacco use. This study aims to explore the potential link between smoking and TB infection using baseline survey data of a large-scale population-based prospective study in rural China

**Methods:**

Between July 1 and Sept 30, 2013, based on the baseline survey of a population-based, prospective study in rural China, the relationship between smoking and TB infection, assessed by interferon-gamma release assays (IGRA), was investigated among the total study population and only among those smokers, respectively.

**Results:**

A total of 21,008 eligible rural registered residents (≥ 5 years old) from 4 rural sites were included in the analysis. Ever-smokers were more likely to be QuantiFERON-TB Gold In-Tube (QFT) positive than never smokers with an adjusted odds ratio (OR) of 1.34 (95% confidence interval (CI): 1.21–1.49). Among ever smokers, a significant linear dose–response relation was observed between duration of smoking (by years) and QFT positivity (p < 0.001). Stratified analysis suggested that such an association was not influenced by gender and age. Evidence for interaction of smoking status with age was found.

**Conclusions:**

Our results provide further evidence to support smoking might increase host susceptibility to TB infection. Populations under high risk of infection, such as elderly smokers, should be prior to TB infection controlling under a premise of community level intervention.

## Introduction

The epidemics of tobacco smoking and tuberculosis (TB) are colliding, and increasing evidence showed smoking was associated with an increased risk of active TB [1–5]. However, the relation between smoking and TB infection has not been widely studied especially in China [6–7]. In spite of the accumulated evidence demonstrating a causal relationship between smoking and TB infection, whether smoking cessation in a community level could contribute to TB controlling is still being debated. Because most of the published studies were limited to special populations at high risk of TB infection, including prisoners [8–9], migrant workers [10–11], immigrants [12–13], and the homeless [14]. Furthermore, several studies even reported a negative association between cigarette smoking and TB infection [15–17]. Therefore, population-based data is urgent needed. Recently, we reported a causal link between smoking and TB infection in a population-based multicenter prospective study conducted in rural China. The annual risk of TB infection among ever-smokers has been found to be 1.53 fold higher as compared to never-smokers [18]. However, further more detailed analysis in the impact of smoking on TB infection was limited due to small sample size of the observed recently infected cases.

Therefore, this study aimed to investigate the association between smoking and TB infection in a community level based on the baseline survey of a large-scale population-based prospective study in rural China.

## Materials and methods

### Study participants

This cross-sectional study was based on the baseline survey of a multicenter prospective cohort study in rural China conducted between July 1st and September 30th, 2013, which has been reported in detail in elsewhere [19]. Registered rural residents at the four study sites were the target populations of the present and previous study. The study sites were selected based on a wide range of local TB epidemiology, economic conditions and geographic diversity (S1 Table) [19]. The inclusion criteria of the study participants were: resident population aged 5 years and above (birth prior to 1 June, 2008); resident population (more than 6 months’ residence in study site in the past year); provision of voluntary written informed consent. The exclusion criteria were suspected or current active tuberculosis (According to WHO guidelines, bacteriological confirmed TB cases (positive for sputum-smear or/and culture or/and X-pert MTB/RIF) or clinically diagnosed case of TB (cases diagnosed on the basis of chest digital radiography abnormalities without laboratory confirmation) were defined as active TB case), self-reported history of tuberculosis, and pregnancy.

### Determination of TB infection

TB infection was tested using both tuberculin skin test (TST) and interferon-γ release assay (IGRA). IGRA result was used to determine TB infection status in the present study because our previous results proved TST results were affected by several factors including age, Bacillus Calmette-Guerin (BCG) vaccination and exposure to non-tuberculosis mycobacterium. QuantiFERON-TB Gold In-Tube (QFT, QIAGEN, USA), a commercial IGRA, was performed as recommended by the manufacturer using a cutoff value of ≥ 0.35 IU/ml.

### Data collection

The study protocol was approved by the ethics committees of the Institute of Pathogen Biology, Chinese Academy of Medical Sciences (No: IPB-2013-5). Upon explanation of the study protocol, written informed consent was obtained from the participant or the legal guardian for all study participants. For each study participant, socio-demographic information was collected by a standardized questionnaire administered by trained interviewers. The questionnaire contained items on demographic characteristics (age, gender, educational level, and household per capita income (RMB)), alcohol consumption, cigarette smoking, history of reported tuberculosis disease, history of type 2 diabetes mellitus (T2DM) (With a self-reported history and/or fast blood glucose higher than 7.0 mmol/L at baseline examination) and history of close contact with a patient with tuberculosis. The following questions included items on smoking conditions: (1) Smoking status: never smoked (smoked less than 5 cigarettes per month or never smoked) and ever smoked (smoked 5 or more cigarettes per month). Ever smokers were further categorized as current smokers and former smokers. The information of duration of abstaining from smoking was collected. (2) Age of smoking onset. (3) Type of cigarette smoking. Two types of cigarette smoking are prevalent in rural China: filter tip cigarettes and sun-cured tobacco without filter tip. The latter was self-made by dried tobacco leaf rolling in a blank paper. (4) Duration of smoking by years and number of cigarettes per day: These exposure were converted into categorical variables for analysis (≤ 10 years, 10–20 years, 20–30 years, 30–40 years, 40–50 years, > 50 years) and (1–5, 6–10, 11–19, and ≥ 20 cigarettes per day), respectively. Body mass index (BMI) was calculated as weight over height squared (kg/m^2^), and was further categorized as underweight (< 18.5 kg/m^2^), normal weight (18.5 to 24.0 kg/m^2^), overweight (24.0 to 28.0 kg/m^2^), or obese (≥ 28.0 kg/m^2^) [20].

### Statistical analysis

Data analyses were performed using SAS 9.2 (SAS Institute Inc., NC, USA). The Pearson’s χ^2^ test was used to compare the categorical variables. Variables with p < 0.05 in the univariate analysis were all included in the multivariable models based on our previous hypothesis Stepwise multiple logistic regression analysis was used to identify the variables independently associated with QFT positivity, the significance level for the variables stayed in the model was 0.05. The associations between TB infection and self-reported history of cigarette smoking were assessed by means of odds ratios (OR) and 95% confidence intervals (CI). Cochran–Armitage (chi-square) tests were used to explore TB infection trends with cigarette intensity. Evidence for interaction of smoking status with gender, age, BMI and TB contact history was assessed using the likelihood ratio test (LRT) by comparing logistic regression models with and without an interaction term. In additional sensitivity analyses, the association between TB infection and self-reported history of cigarette smoking was evaluated after excluding 5–19 years old people.

## Results

A total of 21,022 eligible participants from four study sites completed baseline survey, 21,008 of them were included in this study after excluding 14 with missing data on smoking. Basic characteristics of the study population with respect to smoking status were shown in Table 1. In total, more than half (53.71%, 11,284/21,008) were females and the age ranged from 5 to 99 years with a median age 46 years (interquartile range [IQR]: 27–59 years). Among the study participants, 24.73% (5195/21008) were ever-smokers (4,835 (24.17%) were current-smokers, 360 (1.80%) were former-smokers), and 15,813 (75.27%) had never smoked. Ever-smokers reported higher educational levels and household incomes than never-smokers. BMI distribution showed that 2089 (40.21%) ever- and 5558 (35.15%) never smokers were overweight or obese. Ever smokers (53.38%, 2773/5195) were more likely to drink alcohol than never-smokers (7.70%, 1,217/15,813) (p < 0.001). 235 (4.53%) ever- and 579 (3.66%) never- smokers had a history of close contact with TB patients, respectively. 6.74% (350/5195) ever smokers and 4.22% (667/15813) never smokers had a self-reported history of T2DM or with a baseline fast blood glucose level ≥ 7.0 mmol/L. The QFT positivity for ever-smokers and never-smokers was 28.26% (1468/5195) and 15.72% (2,486/15,813), respectively (p < 0.001).

**Table 1:** Characteristics of the total study population by smoking status.

Variables	Total^†^ (N = 21008)	%	Ever-smoker (n = 5195)	%	Never-smoker (n = 15813)	%	p for χ^2^ test^‡^
**Gender**							<0.001
Male	9724	46.29	5061	97.42	4663	29.49	
Female	11284	53.71	134	2.58	11150	70.51	
**Age (years)**							<0.001
5–19	3556	16.93	46	0.89	3510	22.20	
20–29	2053	9.77	514	9.89	1539	10.53	
30–39	2181	10.38	599	11.53	1582	12.06	
40–49	4648	22.12	1304	25.10	3344	21.15	
50–59	3612	17.19	1170	22.52	2442	15.44	
60–69	3136	14.93	1060	20.40	2076	13.13	
≥70	1822	8.67	502	9.66	1320	8.35	
Median (inter quartile range)	46 (27–59)		50 (41–62)		44 (23–58)		
**Highest education level**							<0.001
Primary school or lower	11172	53.18	2236	43.04	8936	56.51	
Middle school	6882	32.76	2198	42.31	4684	29.62	
High school	2301	10.95	650	12.51	1651	10.44	
College or higher	653	3.11	111	2.14	542	3.43	
**BMI (kg/m**^**2**^**)**							<0.001
<18.5	3278	15.60	278	5.35	3000	18.97	
≥18.5-<24.0	10082	47.99	2828	54.44	7254	45.88	
≥24.0-<28.0	5596	26.64	1542	29.68	4054	25.64	
≥28.0	2051	9.76	547	10.53	1504	9.51	
**Household per capita income (RMB)**							<0.001
<6000	13411	63.84	3178	61.17	10233	64.72	
≥6000	7596	36.16	2017	38.83	5579	35.28	
**Alcohol drinking**							<0.001
No	17017	81.01	2422	46.62	14595	92.30	
Yes	3990	18.99	2773	53.38	1217	7.70	
**TB contact history**							0.005
No	20185	96.12	4958	95.47	15227	96.34	
Yes	814	3.88	235	4.53	579	3.66	
**History of T2DM**							<0.001
Yes	1017	4.84	350	6.74	667	4.22	
No	19911	95.16	4845	93.26	15146	95.78	
**QFT test**							<0.001
Positive	3954	18.82	1468	28.26	2486	15.72	
Negative	16454	78.32	3624	69.76	12830	81.14	
Indeterminate	600	2.86	103	1.98	497	3.14	

Abbreviations: BMI = body mass index; IQR = interquartile range; QFT = QuantiFERON-TB Gold In-Tube; T2DM = type 2 diabetes mellitus; TB = tuberculosis.

^†^Sum might not always be in total because of missing data. Frequency of missing data did not differ significantly between smoking statuses.

^‡^ P values refer to the comparison of different variables between ever- and never-smokers.

**Fig 1:**
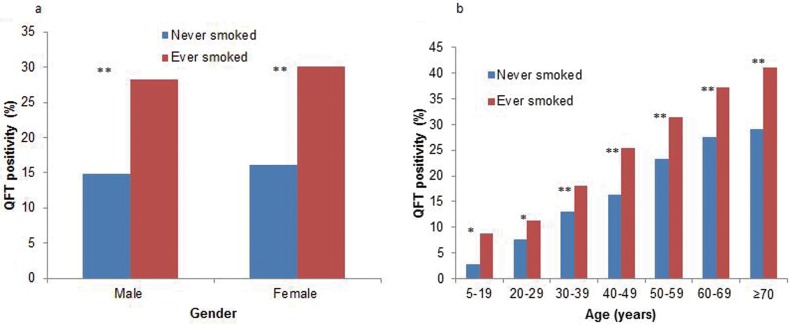
**Distribution of QFT positivity among ever-smokers and never-smokers by smoking status, gender (1A) and age (1B).** After classified by gender and age groups, ever-smokers were still more likely to be QFT positivity than never-smokers (*: p < 0.05, **: p < 0.01). Abbreviations: QFT = QuantiFERON-TB Gold In-Tube.

Univariate and multivariate analysis of QFT positivity were conducted firstly among total study population (Table 2). Factors significantly associated with QFT positivity were male sex, increasing age, a BMI of 28.0 kg/m^2^ or more, ever smoked and history of close contact with TB patients. The risk of QFT positivity among ever-smokers was 1.34 times higher than never-smokers (95% CI: 1.21–1.49) in multivariate analysis. After classified by gender and age groups, ever-smokers were still more likely to be QFT positive than never smokers as depicted in Fig 1.

**Table 2:** Identification of the factors independently associated with QFT positivity in the total study population.

Variables	QFT positivity(n/N^†^)	%	p for χ^2^ test	Adjusted OR^#^ (95% CI)
**Gender**				
Female	1832/10863	16.86	<0.001	Reference
Male	2122/9545	22.23		1.26 (1.14, 1.39)
**Age (years)**				
5–19	100/3485	2.87	<0.001	Reference
20–29	175/2009	8.71		2.72 (2.07, 3.56)
30–39	313/2114	14.81		4.83 (3.73, 6.25)
40–49	876/4510	19.42		6.64 (5.22, 8.45)
50–59	934/3478	26.85		9.97 (7.83, 12.69)
60–69	966/3047	31.70		12.65 (9.95, 16.07)
≥70	590/1765	33.43		14.27 (11.19, 18.21)
**Education level**				
Primary school or lower	2236/10796	20.71	<0.001	
Middle school	1275/6714	18.99		
High school	375/2259	16.60		
College or higher	68/639	10.64		
**Household per capita income (RMB)**				
<6000	2512/12978	19.36	0.924	
≥6000	1442/7429	19.41		
**BMI (kg/m**^**2**^**)**				
<18.5	230/3203	7.18	<0.001	0.86 (0.73, 1.02)
≥18.5-<24.0	2002/9762	20.51		Reference
≥24.0-<28.0	1257/5444	23.09		1.06 (0.98, 1.16)
≥28.0	465/1998	23.27		1.15 (1.02, 1.29)
**Smoking status**				
Never smoked	2486/15316	16.23	<0.001	Reference
Ever smoked	1468/5092	28.83		1.34 (1.21, 1.49)
**Alcohol drinking**				
No	2957/16486	17.94	<0.001	
Yes	997/3921	25.21		
**TB contact history**				
No	3722/19618	18.97	<0.001	Reference
Yes	231/781	29.58		1.62 (1.37, 1.90)
**History of T2DM**				
No	3689/19427	18.99	<0.001	
Yes	265/981	27.01		

Abbreviations: BMI = body mass index; CI = confidence interval; OR = odds ratio; QFT = QuantiFERON-TB Gold In-Tube; T2DM = type 2 diabetes mellitus; TB = tuberculosis.

^†^ Participants with indeterminate results were not included in this analysis. Sum might not always be in total because of missing data.

^# ^Adjusted for variables with p < 0.05 in univariate analysis by stepwise selection. Sex and age kept in the model.

**Fig 2:**
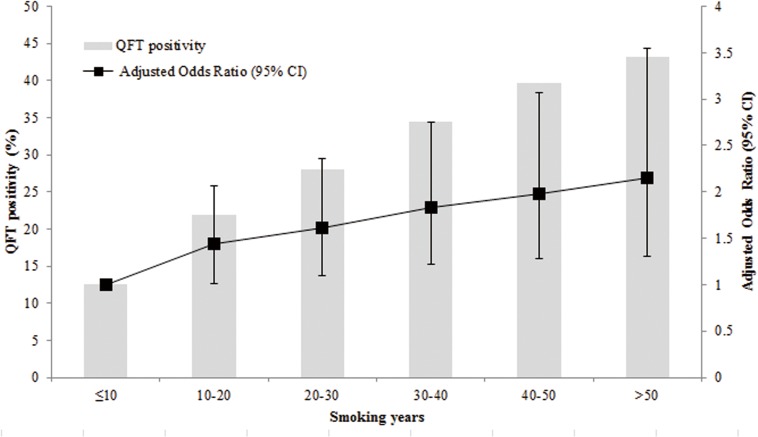
Association of QFT positivity with smoking years among smokers. Multiple logistic regression analysis was conducted to assess the association of smoking years with QFT positivity. Variables with p < 0.05 in univariate analysis were included in the model. Gender, age, education level, history of close contact with tuberculosis patients, alcohol drinking, number of cigarettes smoked per day were adjusted. Adjusted odds ratio (ORs) and the 95% confidence intervals (CIs) were given in the figure. Abbreviations: CI = confidence interval; QFT = QuantiFERON-TB Gold In-Tube.

Factors associated with QFT positivity among smokers were shown in Table 3. An increasing TB infection risk was observed to be positively related to ages of 60 years or older, history of close contact with TB patients and duration of smoking years. In addition, as shown in Fig 2, a significant dose–response relation between QFT positivity and duration of smoking years was observed among smokers (p trend = 0.001).

**Table 3:** Association analysis for QFT positivity among smokers.

Variables	N^†^	%	p for χ^2^ test	Adjusted OR^#^(95% CI)
**Gender**				
Female	41/131	31.30	0.528	Reference
Male	1427/4961	28.76		0.91 (0.62.1.35)
**Age (years)**				
5–19	4/46	8.70	<0.001	Reference
20–29	58/506	11.46		1.30 (0.45,3.75)
30–39	108/590	18.31		1.72 (0.58,5.09)
40–49	331/1285	25.76		2.27 (0.76,6.72)
50–59	367/1139	32.22		2.74 (0.91,8.19)
60–69	394/1033	38.14		3.25 (1.08,9.79)
≥70	206/493	41.78		3.53 (1.15, 10.85)
**Education level**				
Primary school or lower	731/2179	33.55	<0.001	
Middle school	560/2162	25.90		
High school	155/643	24.11		
College or higher	22/108	20.37		
**Household per capita income (RMB)**				
<6000	924/3108	29.74	0.076	
≥6000	544/1984	27.42		
**BMI (kg/m**^**2**^**)**				
18.5–24.0	814/2766	29.43	0.520	
<18.5	71/276	25.72		
24.0–28.0	436/1515	28.78		
≥28.0	147/535	27.48		
**Alcohol drinking**				
No	732/2358	31.04	0.001	
Yes	736/2734	26.92		
**TB contact history**				
No	1381/4862	28.40	0.002	Reference
Yes	87/228	38.16		1.48 (1.12,1.97)
**History of T2DM**				
No	1352/4751	28.46	0.029	
Yes	116/341	34.02		
**Smoking status**				
Former smoker	101/352	28.69	0.953	
Current smoker	1367/4740	28.84		
**Cigarette type**				
Without filter	108/324	33.33	0.064	
With filter	1360/4768	28.52		
**Onset of cigarette smoking (years)**				
<18	279/927	30.10	0.346	
≥18	1189/4165	28.55		
**Duration of smoking (years)**				
≤10	96/753	12.75	<0.001	Reference
10–20	199/891	22.33	p for trend<0.001	1.45 (1.02, 2.06)
20–30	366/1305	28.05		1.65 (1.14, 2.40)
30–40	392/1138	34.45		1.90(1.28, 2.82)
40–50	294/726	40.50		2.09 (1.36, 3.19)
>50	121/279	43.37		2.26 (1.38, 3.69)
**Number of cigarettes per day**				
1–5	126/507	24.85	0.009	
5–10	182/683	26.65	p for trend<0.001	
10–19	525/1912	27.46		
≥20	635/1990	31.91		

Abbreviations: BMI = body mass index; CI = confidence interval; OR = odds ratio; QFT = QuantiFERON-TB Gold In-Tube; T2DM = type 2 diabetes mellitus; TB = tuberculosis.

^† ^Indeterminate results had been excluded from the analysis. Sum might not always be in total because of missing data.

^# ^Adjusted for variables with p < 0.05 in univariate analysis by stepwise selection. Sex and age kept in the model.

As shown in Table 4, we found evidence of modification effect by age (p for interaction < 0.001) with an OR ranged from 3.44 (1.16, 10.16) for those aged less than 19 to an OR of 1.31 (1.10, 1.55) for those older than 60. We did not observe a modification effect of gender (p for interaction, 0.842), BMI (p for interaction, 0.838) or history of TB contacts (p for interaction, 0.931). In additional sensitivity analyses, excluding 3556 subjects who were 5–19 years old did not materially alter the results (S2 and S3 Tables).

**Table 4:** The association of QFT positivity and smoking status by age, gender and TB contact history among total study populations.

Variables^&^		QFT positivity (n/N)	%	Adjusted OR (95%CI)
**Gender and smoking status**^†^				
Female	Never-smoker	1791/10732	16.69	Reference
	Ever-smoker	41/131	31.30	1.70 (1.16, 2.50)
Male	Never-smoker	695/4584	15.16	Reference
	Ever-smoker	1427/4961	28.76	1.45 (1.30, 1.61)
p for likelihood ratio test				0.842
**Age and smoking status**^‡^				
5–19 years	Never-smoker	96/3439	2.79	Reference
	Ever-smoker	4/46	8.70	3.44 (1.16, 10.16)
20–39 years	Never-smoker	322/3027	10.64	Reference
	Ever-smoker	166/1096	15.15	1.40 (1.07, 1.85)
40–59 years	Never-smoker	1112/5564	19.99	Reference
	Ever-smoker	698/2424	28.80	1.32 (1.13, 1.55)
≥ 60 years	Never-smoker	956/3286	29.09	Reference
	Ever-smoker	600/1526	39.32	1.31 (1.10,1.55)
p for likelihood ratio test				0.002
**TB contact history and smoking status**^**#**^				
No	Never-smoker	2341/14756	15.86	Reference
	Ever-smoker	1381/4862	28.40	1.49 (1.34, 1.66)
Yes	Never-smoker	144/553	26.04	Reference
	Ever-smoker	87/228	38.16	1.32 (0.84, 2.06)
p for likelihood ratio test				0.931
BMI and smoking status^*^				
<28.0(kg/m^2^)	Never-smoker	2168/13853	15.65	Reference
	Ever-smoker	1321/4557	28.99	1.47 (1.32, 1.97)
≥28.0 (kg/m^2^)	Never-smoker	318/1463	21.74	Reference
	Ever-smoker	147/535	27.48	1.55 (1.11, 2.15)
p for likelihood ratio test				0.838

Abbreviations: BMI = Body mass index; CI = confidence interval; OR = odds ratio; QFT = QuantiFERON-TB Gold In-Tube; TB = tuberculosis. Participants with indeterminate results were not included in this analysis. Sum might not always be in total because of missing data.

^&^ Variables independently associated with QFT positivity identified in Table 2 were considered in this analysis.

^†^Adjusted for age, BMI and TB contact history.

^‡^ Adjusted for gender, BMI and TB contact history.

^**#**^ Adjusted for age, BMI and gender.

^*^ Adjusted for age, TB contact history and gender.

## Discussion

In this large cross-sectional analysis among 21,008 participants in rural China, ever smoking was found to be significantly associated with TB infection. Among smokers, smoking years was identified as an independent risk factor which was strongly related to TB infection in a dose-response manner. Our results suggested that the development of smoking bans in China might benefit TB control as well. High risk populations for TB infection, such as smoking elderly found in this study, might be potential target populations for TB infection monitoring and management in China.

The World Health Organization published a monograph to announce the integration of tobacco control into TB programs in 2007 [21]. However, the impact of smoking on TB infection has not been clearly estimated. China not only has the greatest number of smokers but also carry some of the highest smoking rates. The geographic overlap between rural areas of China with a higher prevalence of cigarette smoking and regions with higher prevalence of latent and active TB is striking and complex, but the potential for significant public health impact is unparalleled. Also, based on the results of our previous study, 13.5%-19.8% of participants were QFT positive in rural China. This may represent an enormous reservoir for tuberculosis transmission and rural areas are a logical and strategic focus for intervention. The results in our investigation were consistent with several studies in population survey settings such as South Africa [22], America [23] which found a positive link between smoking and TST positivity. More importantly, our results might be more robust after excluding the potential interferences from BCG vaccination and non-tuberculosis mycobacteria (NTM) by using IGRA. However, by contrast with the increasing risk of TB infection among smokers in our study, such a relation was not found in the study performed in Zambia and South Africa [15, 17]. The different characteristics of the study populations might account for the disparity. Both of the studies examined the influence of smoking on QFT results in human immunodeficiency virus (HIV) infections. The decreased sensitivity of QFT test in immune-suppressed individuals underestimated the prevalence of TB infection and might finally lead to bias [24]. In addition, smoking habit might be different among populations at higher risk of HIV infection and might be changed after HIV infection acquisition. Therefore, we should take care to generalize the findings from the specific population to the general population.

This study showed that there was a positive dose-response relationship between smoking years and TB infection. The adverse effects of cigarette smoking on pulmonary immunity might explain the susceptibility of smoking population to TB infection [25]. Cigarette smoking may attenuate host defense mechanisms by preventing expansion and activation of pathogen-specific CD4+ T-cells and reducing the numbers of IFN-γ-producing adenoid-specific CD4+ and CD8+ T-cells [26]. Cumulative stimulation of cigarette smoking might selectively down-regulates the production of IL-12 and TNF-α. Simultaneously, nicotine could turn off production of TNF-α by macrophages while leaving the secretion of IL-10 intact [27]. However, it is worth concern that the aged population probably had longer duration and/or higher intensity in cigarette smoking and the lung structure impairment in aged patients may also increase their susceptibilities to TB infection. A study from Taiwan found evidence of effect modification by age because the effect of current smoking on active TB disappeared among those aged 65 or older [28]. Evidence of effect modification between age and smoking were also found in our study. Unlike the study from Taiwan, the prevalence of TB infection increased from 2.79% among < 20 years’ never-smokers to 39.32% among ≥ 60 years’ ever-smokers (classified by 20 years). The effect of smoking on TB infection was always statistically significant although it decreased with age, which hinted the necessity of advocating smoking cessation among older smokers. Otherwise, in contrast to several studies [8, 11], our study failed to find a correlation between numbers of cigarettes smoked per day and TB infection among smoking populations. There are two explanations for this discrepancy. Firstly, our large sample size gives us the opportunity to stratify this analysis among smokers, but the former studies mainly used never-smokers as control. Second, co-linearity might exist between years of smoking and numbers of cigarettes smoked due to the potential tendency for smokers to smoke more and more as times went on. We verified our hypotheses by analyzing the effect of numbers of cigarettes smoked per day in whole population. Comparing with never-smokers, a dose-effect association was observed with OR ranged from 1.19 to 1.44 after controlling for age, gender, BMI and close contact history (S4 Table). However, the associations were non-significant when adding smoking years into the model. But the effect of smoking years was still significant in such a model. Therefore, smoking years might be a better indicator to reflect the impact of cigarette intensity on TB infection.

Our findings should be interpreted with consideration of the following limitations. First, the cross-sectional analysis could not absolutely determine the causal link between cigarette smoking and TB infection. During the follow-up period of the study, the relationship between smoking and the risk of TB infection acquisition will be further studied to clarify this important issue. Second, smoking status was self-reported rather than determined by biochemical methods. A previous systematic review showed trends of underestimation when smoking prevalence was based on self-report compared to cotinine-assessed [29]. Although a serious of related questions on smoking were collected which might reduce the possibility of such information bias, but we couldn’t exclude it completely. Thirdly, Second Hand Smoke (SHS) exposure or passive smoking [30] and air pollution [6] had been reported to be associated with elevated risk for TB infection. While we failed to clarify this effect as we didn’t collect related information.

## Conclusions

Despite of the limitations, our findings support that cigarette smoking was independently associated with increased risk of TB infection. More attention should be attached to smokers, especially elderly smokers, as one potential target population for TB infection controlling in rural China. Reinforced programs to reduce cigarette use or development of regimen on tobacco cessation might contribute to lighten the burden of TB infection. In addition, active case finding among populations with specific risks such as smokers [31], close contacts [32], diabetes [33] should be strengthened as well.

## Supporting information

S1 Table Population sampling among the study sites at the baseline survey in 2013. (DOC)

S2 Table Association analyses for QFT positivity among study population aged 20 years or older. (DOC)

S3 Table Association analysis for QFT positivity among smokers aged 20 years or older. (DOC)

S4 Table Multivariate analysis of QFT positivity classified by number of cigarettes per day. (DOC)
