# Supplementary material for: A dose-response relationship of smoking with tuberculosis infection: A cross-sectional study among 21008 rural residents in China
Source: PLoS One. 2017 Apr 6;12(4):e0175183. doi: 10.1371/journal.pone.0175183 (PMC5383252; doi:10.1371/journal.pone.0175183)
Supplement: S1 Table — (DOC) [file pone.0175183.s001.doc]

**S1 Table. Population sampling among the study sites at the baseline survey in 2013**

|  | **Site A** | **Site B** | **Site C** | **Site D** |
| --- | --- | --- | --- | --- |
| Geographic location | Eastern China, plains | Central China, plains | Western China, hills | Western China, basin |
| [Income per capita](http://dict.cn/income per capita) in 2012 | 14,396 RMB | 11,326 RMB | 7,800 RMB | 4,427 RMB |
| Proportion of migrant worker population in 2012 | 10.4% | 5.0% | 23.5% | 11.8% |
| Reported incidence of active tuberculosis in 2010 (per 100,000 population) | 53.5 | 91.3 | 105.6 | 81.9 |
