# Supplementary material for: A dose-response relationship of smoking with tuberculosis infection: A cross-sectional study among 21008 rural residents in China
Source: PLoS One. 2017 Apr 6;12(4):e0175183. doi: 10.1371/journal.pone.0175183 (PMC5383252; doi:10.1371/journal.pone.0175183)
Supplement: S2 Table — (DOC) [file pone.0175183.s002.doc]

**S2 Table. Association analyses for QFT positivity among study population aged 20 years or older**

| **Variables** | **QFT positivity n/N^†^** | **%** | **p for χ^2^ test** | **Adjusted OR**^‡^ **(95% CI)** |
| --- | --- | --- | --- | --- |
| **Gender** |  |  |  |  |
| Female | 1785/9235 | 19.33 | <0.001 | Reference |
| Male | 2069/7688 | 26.91 |  | 1.28 (1.16,1.42) |
| **Age (years)** |  |  |  |  |
| 20-29 | 175/2009 | 8.71 | <0.001 | Reference |
| 30-39 | 313/2114 | 14.81 |  | 1.78 (1.46, 2.16) |
| 40-49 | 876/4510 | 19.42 |  | 2.44 (2.05, 2.91) |
| 50-59 | 934/3478 | 26.85 |  | 3.67 (3.08,4.36) |
| 60-69 | 966/3047 | 31.70 |  | 4.65 (3.91, 5.54) |
| ≥70 | 590/1765 | 33.43 |  | 5.26 (4.38, 6.33) |
| **Education level** |  |  |  |  |
| Primary school or lower | 2175/8563 | 25.40 | <0.001 |  |
| Middle school | 1259/5980 | 21.05 |  |  |
| High school | 355/1792 | 19.81 |  |  |
| College or higher | 65/588 | 11.05 |  |  |
| **Household per capita income (RMB)** |  |  |  |  |
| <6000 | 2437/10358 | 23.53 | 0.003 |  |
| ≥6000 | 1417/6564 | 21.59 |  |  |
| **BMI (kg/m^2^)** |  |  |  |  |
| <18.5 | 159/833 | 19.09 | 0.016 | 0.81 (0.68, 0.98) |
| ≥18.5-<24.0 | 1978/8827 | 22.41 |  | Reference |
| ≥24.0-<28.0 | 1254/5318 | 23.58 |  | 1.06 (0.98, 1.15) |
| ≥28.0 | 463/1944 | 23.82 |  | 1.14 (1.02, 1.29) |
| **Smoking status** |  |  |  |  |
| Never smoker | 2390/11877 | 20.12 | <0.001 | Reference |
| Ever smoker | 1464/5046 | 29.01 |  | 1.32 (1.19, 1.47) |
| **Alcohol drinking** |  |  |  |  |
| No | 2859/13065 | 21.88 | <0.001 |  |
| Yes | 995/3857 | 25.80 |  |  |
| **TB contact history** |  |  |  |  |
| No | 3633/16213 | 22.41 | <0.001 | Reference |
| Yes | 220/701 | 31.38 |  | 1.53 (1.29, 1.81) |
| **History of type 2 diabetes** |  |  |  |  |
| No | 3591/15954 | 22.51 | <0.001 |  |
| Yes | 263/969 | 27.14 |  |  |

Abbreviations: BMI=body mass index; CI=confidence interval; OR=odds ratio; QFT=QuantiFERON-TB Gold In-Tube; TB=tuberculosis.

^†^ Participants with indeterminate results were not included in this analysis. Sum might not always be in total because of missing data

^‡^ Adjusted for variables with p < 0.05 in univariate analysis by stepwise selection. Sex and age kept in the model
