# Supplementary material for: A dose-response relationship of smoking with tuberculosis infection: A cross-sectional study among 21008 rural residents in China
Source: PLoS One. 2017 Apr 6;12(4):e0175183. doi: 10.1371/journal.pone.0175183 (PMC5383252; doi:10.1371/journal.pone.0175183)
Supplement: S3 Table — (DOC) [file pone.0175183.s003.doc]

**S3 Table. Association analysis for QFT positivity among smokers aged 20 years or older**

| **Variables** | **N^†^** | **%** | **p for χ^2^ test** | **Adjusted OR**^‡^ **(95% CI)** |
| --- | --- | --- | --- | --- |
| **Gender** |  |  |  |  |
| Female | 41/131 | 31.30 | 0.559 | Reference |
| Male | 1423/4915 | 28.95 |  | 0.91 (0.62.1.34) |
| **Age (years)** |  |  |  |  |
| 20-29 | 58/506 | 11.46 | <0.001 | Reference |
| 30-39 | 108/590 | 18.31 |  | 1.33 (0.87, 2.02) |
| 40-49 | 331/1285 | 25.76 |  | 1.75 (1.14, 2.68) |
| 50-59 | 367/1139 | 32.22 |  | 2.11 (1.34, 3.31) |
| 60-69 | 394/1033 | 38.14 |  | 2.50 (1.56, 4.00) |
| ≥70 | 206/493 | 41.78 |  | 2.72 (1.63, 4.55) |
| **Education level** |  |  |  |  |
| Primary school or lower | 731/2176 | 33.59 | <0.001 |  |
| Middle school | 558/2133 | 26.16 |  |  |
| High school | 153/629 | 24.32 |  |  |
| College or higher | 22/108 | 20.37 |  |  |
| **Household per capita income (RMB)** |  |  |  |  |
| <6000 | 921/3073 | 29.97 | 0.061 |  |
| ≥6000 | 543/1973 | 27.52 |  |  |
| **BMI (kg/m^2^)** |  |  |  |  |
| 18.5–24.0 | 69/267 | 25.84 | 0.538 |  |
| <18.5 | 812/2744 | 29.59 |  |  |
| 24.0–28.0 | 436/1505 | 28.97 |  |  |
| ≥28.0 | 147/530 | 27.74 |  |  |
| **Alcohol drinking** |  |  |  |  |
| No | 730/2344 | 31.14 | 0.002 |  |
| Yes | 734/2702 | 24.17 |  |  |
| **TB contact history** |  |  |  |  |
| No | 1377/4817 | 28.59 | 0.002 | Reference |
| Yes | 87/227 | 38.33 |  | 1.49 (1.12,1.98) |
| **History of type 2 diabetes** |  |  |  |  |
| No | 116/341 | 28.65 | 0.035 |  |
| Yes | 1348/4705 | 34.02 |  |  |
| **Smoking status** |  |  |  |  |
| Former smoker | 101/352 | 28.69 | 0.891 |  |
| Current smoker | 1363/4694 | 29.04 |  |  |
| **Cigarette type** |  |  |  |  |
| Without filter |  |  | 0.064 |  |
| With filter |  |  |  |  |
| **Onset of cigarette smoking (years)** |  |  |  |  |
| <18 | 275/887 | 31.00 | 0.150 |  |
| ≥18 | 189/4159 | 28.59 |  |  |
| **Duration of smoking (years)** |  |  |  |  |
| ≤10 | 89/691 | 12.88 | <0.001 | Reference |
| 10-20 | 189/864 | 21.88 | p for trend<0.001 | 1.45 (1.02, 2.06) |
| 20-30 | 360/1286 | 27.99 |  | 1.65 (1.14, 2.40) |
| 30-40 | 391/1135 | 34.45 |  | 1.90(1.28, 2.82) |
| 40-50 | 299/755 | 39.60 |  | 2.09 (1.36, 3.19) |
| >50 | 136/315 | 43.17 |  | 2.26 (1.38, 3.70) |
| **Number of cigarettes per day** |  |  |  |  |
| 0-5 | 124/489 | 25.36 | 0.002 |  |
| 5-10 | 181/671 | 26.97 | p for trend=<0.0004 |  |
| 10-19 | 524/1899 | 27.59 |  |  |
| ≥20 | 635/1987 | 31.96 |  |  |

Abbreviations: BMI=body mass index; CI=confidence interval; OR=odds ratio; QFT=QuantiFERON-TB Gold In-Tube; TB=tuberculosis.

^†^ Indeterminate results had been excluded from the analysis. Sum might not always be in total because of missing data

^‡^ Adjusted for variables with p < 0.05 in univariate analysis by stepwise selection. Sex and age kept in the model.
