# Supplementary material for: A dose-response relationship of smoking with tuberculosis infection: A cross-sectional study among 21008 rural residents in China
Source: PLoS One. 2017 Apr 6;12(4):e0175183. doi: 10.1371/journal.pone.0175183 (PMC5383252; doi:10.1371/journal.pone.0175183)
Supplement: S4 Table — (DOC) [file pone.0175183.s004.doc]

**S4 Table Multivariate analysis of QFT positivity classified by number of cigarettes per day**

| **Number of cigarettes per day** | **Adjusted OR^†^ (95%CI)** |
| --- | --- |
| Never smokers | Reference. |
| < 5 | 1.18 (0.95-1.48) |
| 5-9 | 1.31 (1.08-1.59) |
| 10-19 | 1.29 (1.13-1.47) |
| ≥ 20 | 1.46 (1.28-1.66) |

Abbreviations: BMI=body mass index; CI=confidence interval; OR=odds ratio; QFT=QuantiFERON-TB Gold In-Tube.

**^†^** Adjusted for gender, age, BMI and close contact history.
